# Bifidobacterium dentium Fortifies the Intestinal Mucus Layer via Autophagy and Calcium Signaling Pathways

**DOI:** 10.1128/mBio.01087-19

**Published:** 2019-06-18

**Authors:** Melinda A. Engevik, Berkley Luk, Alexandra L. Chang-Graham, Anne Hall, Beatrice Herrmann, Wenly Ruan, Bradley T. Endres, Zhongcheng Shi, Kevin W. Garey, Joseph M. Hyser, James Versalovic

**Affiliations:** a Department of Pathology and Immunology, Baylor College of Medicine, Houston, Texas, USA; b Department of Pathology, Texas Children’s Hospital, Houston, Texas, USA; c Department of Molecular Virology and Microbiology, Baylor College of Medicine, Houston, Texas, USA; d Department of Pharmacy Practice and Translational Research, University of Houston College of Pharmacy, Houston, Texas, USA; Polish Academy of Sciences, Institute of Immunology and Experimental Therapy; CIML

**Keywords:** acetate, bifidobacteria, epithelium, GABA, glycans, goblet cells, gut microbes, intestine, Muc2, mucins, probiotics

## Abstract

Much remains unknown about how the intestinal microbiome interfaces with the protective intestinal mucus layer. Bifidobacterium species colonize the intestinal mucus layer and can modulate mucus production by goblet cells. However, select *Bifidobacterium* strains can also degrade protective glycans on mucin proteins. We hypothesized that the human-derived species Bifidobacterium dentium would increase intestinal mucus synthesis and expulsion, without extensive degradation of mucin glycans. *In silico* data revealed that B. dentium lacked the enzymes necessary to extensively degrade mucin glycans. This finding was confirmed by demonstrating that *B. dentium* could not use naive mucin glycans as primary carbon sources *in vitro*. To examine *B. dentium* mucus modulation *in vivo*, Swiss Webster germfree mice were monoassociated with live or heat-killed *B. dentium*. Live *B. dentium*-monoassociated mice exhibited increased colonic expression of goblet cell markers *Krüppel-like factor 4* (*Klf4*), *Trefoil factor 3* (*Tff3*), *Relm*-β, *Muc2*, and several glycosyltransferases compared to both heat-killed *B. dentium* and germfree counterparts. Likewise, live *B. dentium*-monoassociated colon had increased acidic mucin-filled goblet cells, as denoted by Periodic Acid-Schiff-Alcian Blue (PAS-AB) staining and MUC2 immunostaining. *In vitro*, *B. dentium*-secreted products, including acetate, were able to increase MUC2 levels in T84 cells. We also identified that *B. dentium*-secreted products, such as γ-aminobutyric acid (GABA), stimulated autophagy-mediated calcium signaling and MUC2 release. This work illustrates that *B. dentium* is capable of enhancing the intestinal mucus layer and goblet cell function via upregulation of gene expression and autophagy signaling pathways, with a net increase in mucin production.

## Abstract (precis)

Microbe-host interactions in the intestine occur along the mucus-covered epithelium. In the gastrointestinal tract, mucus is composed of glycan-covered proteins, or mucins, which are secreted by goblet cells to form a protective gel-like structure above the epithelium. Low levels of mucin or alterations in mucin glycans are associated with inflammation and colitis in mice and humans. Although current literature links microbes to the modulation of goblet cells and mucins, the molecular pathways involved are not yet fully understood. Using a combination of gnotobiotic mice and mucus-secreting cell lines, we have identified a human-derived microbe, Bifidobacterium dentium, which adheres to intestinal mucus and secretes metabolites that upregulate the major mucin MUC2 and modulate goblet cell function. Unlike other *Bifidobacterium* species, *B. dentium* does not extensively degrade mucin glycans and cannot grow on mucin alone. This work points to the potential of using *B. dentium* and similar mucin-friendly microbes as therapeutic agents for intestinal disorders with disruptions in the mucus barrier.

## INTRODUCTION

The mucus layer is the first point of contact between the gut microbiota and the host. Intestinal mucus is composed primarily of the gel-forming secreted mucin MUC2 synthesized by intestinal goblet cells (1–3). Mucins form homodimers in the endoplasmic reticulum (ER) and are *O-*glycosylated in the Golgi apparatus. Glycosylation is a key step to produce functionally mature mucus, and mucin *O-*linked glycans account for up to 80% of the mucin protein’s molecular weight (4–8). Mature glycosylated mucins are packaged into mucus granules that can be secreted constitutively or released after stimulation by specific agonists (3). The intestinal mucus layer is important for overall health since disruption of this boundary can contribute to intestinal inflammation (1, 9–13). For example, ulcerative colitis (UC) patients have reduced synthesis and secretion of mucins, altered *O-*glycosylation, decreased mucus thickness, and increased bacterial penetration of the mucus barrier (9–11, 14). These findings are reflected in mouse models as well, where a loss of MUC2 proteins or their glycans results in colitis (2, 15–21). Due to their beneficial properties, including mucus modulation, bifidobacteria have been proposed as a potential treatment for human diseases characterized by an impaired mucus layer (22–28). Bifidobacterium strains have been reported to promote remission for UC patients (29, 30), lending credence to the concept of using bifidobacteria as a potential therapeutic agent for mucin-related disorders.

Bifidobacterium species are among the first colonizers of the gastrointestinal (GI) tract (31–39). Although bifidobacteria represent only 3 to 6% of the healthy adult fecal microbiota (40, 41), their presence has been associated with numerous health benefits (29, 30, 42–63). However, the molecular mechanisms that underlie these positive effects, which appear to be relatively strain specific, remain unclear (64, 65). Therefore, it is important to understand which molecular strategies are employed by select species in order to characterize their individual effects on the host. In particular, bifidobacteria are known to adhere to intestinal mucins and colonize the mucus layer of the GI tract (66–68). Close proximity of bacterium and host cells may promote health-mediating effects of bifidobacteria (67–70). Although bifidobacteria modulate MUC2 levels (24–27), several well-characterized *Bifidobacterium* species harbor glycosyl hydrolases which can extensively degrade mucin glycans (7, 71–81). While these mucin-degrading enzymes are likely important in GI niche development, the ability of select bifidobacterial species to degrade mucin glycans may be unfavorable when there is diminished mucin production, such as during colitis. These findings emphasize the need to characterize the nature of the mucin-modulating capacity of *Bifidobacterium* strains. Our model strain of Bifidobacterium dentium was isolated from the feces of healthy infants and adults (38, 82–85) and has been observed in healthy adults at a relative abundance of 0.7% in studies published by the Human Microbiome Project Consortium (86–90). While much work has addressed the effects of several *Bifidobacterium* species on the host, few studies have examined how B. dentium modulates the intestinal environment.

Using gnotobiotic mice, we have identified that *B. dentium* adheres to intestinal mucus and colonizes the mucus layer of the colon. In contrast to other well-characterized *Bifidobacterium* strains which contain numerous mucin-degrading glycosyl hydrolases, *B. dentium* harbors only 4 glycosyl hydrolases involved in mucin degradation. This biochemical feature is reflected by the inability of *B. dentium* to grow with mucin as the sole carbon source. We show that colonization by *B. dentium* is associated with increased *Muc2* expression and MUC2 synthesis, in addition to alterations in glycosyltransferases and terminal glycans. We have positively identified two *B. dentium*-secreted compounds that modulate goblet cells, as follows: acetate, which stimulates MUC2 synthesis, and γ-aminobutyric acid (GABA), which promotes autophagy and calcium mobilization to release stored mucin granules from intestinal goblet cells. This study is among the first to characterize *B. dentium* mucus modulation and points to the role of *B. dentium* as a mucin builder (versus the mucin degraders and mucin maintainers) and a possible therapeutic agent for diseases with disrupted mucus barriers.

## RESULTS

### *B. dentium* adheres to intestinal MUC2.

**FIG 1:**
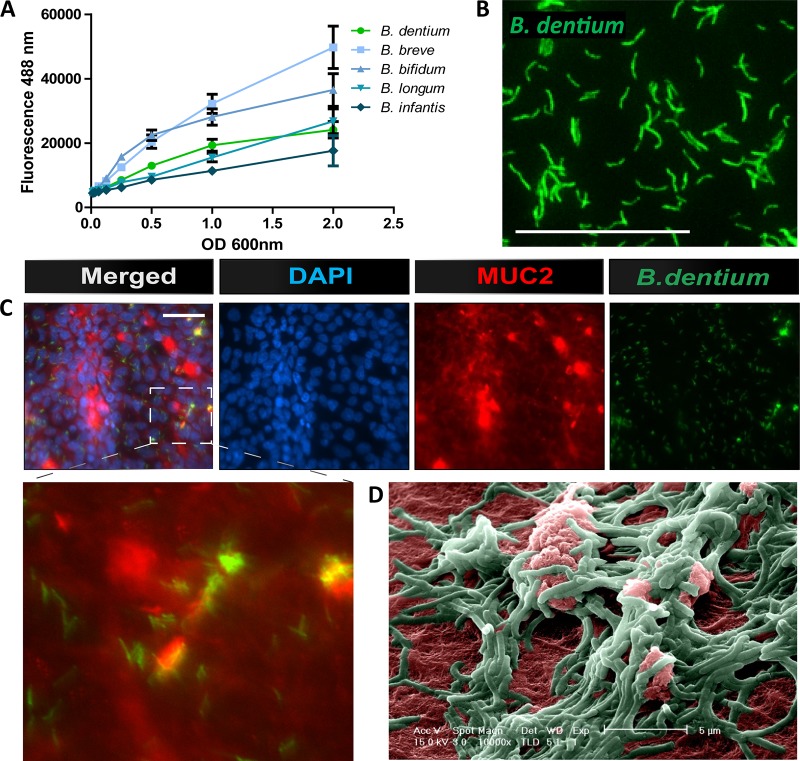
*B. dentium* adheres to intestinal Muc2. (A) CFDA-SE fluorescently tagged *B. dentium, B. breve, B. bifidum*, B. longum subsp. *longum*, and B. longum subsp. *infantis* adhesion to purified germfree mouse cecal MUC2 as denoted by fluorescence (excitation/emission, 488/528 nm) (*n* = 8/group, representative of 3 independent experiments. (B) Representative immunofluorescence images (×600 magnification, scale bar = 50 μm) of CFDA-SE-tagged *B. dentium* adhesion to germfree MUC2. (C). Representative images (×200 magnification, scale bar = 50 μm) of *B. dentium* (green) colocalizes with MUC2 (red) in human LS174T goblet cell by immunostaining; scale bar = 50 μm. DAPI, 4′,6-diamidino-2-phenyindole. (D) Scanning electron microscopy (SEM) images of *B. dentium* (green) and mucus (red) in LS174T cells (color added artificially, scale bar = 5 μm).

Adhesion to the intestinal mucus layer is considered a prerequisite for colonization by mucosa-associated bacteria and represents a selection criterion for probiotic microbes (91). Given the importance of intestinal mucus at the microbe-mammal interface, we sought to identify whether Bifidobacterium dentium could adhere to and modulate intestinal mucins. To determine the adhesion capabilities of *B. dentium* to intestinal mucus, *B. dentium* was fluorescently tagged with carboxyfluorescein diacetate- succinimidyl ester (CFDA-SE) and incubated for 1 h with purified germfree mouse MUC2 at various optical densities (ODs) (Fig. 1A and B). For comparison, we included CFDA-SE-tagged *Bifidobacterium* species that are known to adhere to mucins, including B. breve, B. bifidum, B. longum subsp. longum, and B. longum subsp. infantis (67, 68, 92). *Bifidobacterium* strains varied in their ability to adhere to mucins, with *B. breve* exhibiting the greatest degree of adhesion and B. longum subsp. *infantis* the lowest degree of adhesion. *B. dentium* adhered to MUC2 to a similar degree as B. longum subsp. *longum*, indicating the comparable ability of *B. dentium* to adhere to mouse MUC2. Additionally, *B. dentium* colocalized with MUC2 in the mucus-producing human cell line LS174T, as observed by immunofluorescence and scanning electron microscopy (SEM) (Fig. 1C and D). These data indicate that *B. dentium*, similar to other well-characterized bifidobacteria, is able to adhere to mouse and human MUC2.

### *B. dentium* harbors relatively few mucin-related glycosyl hydrolases.

**FIG 2:**
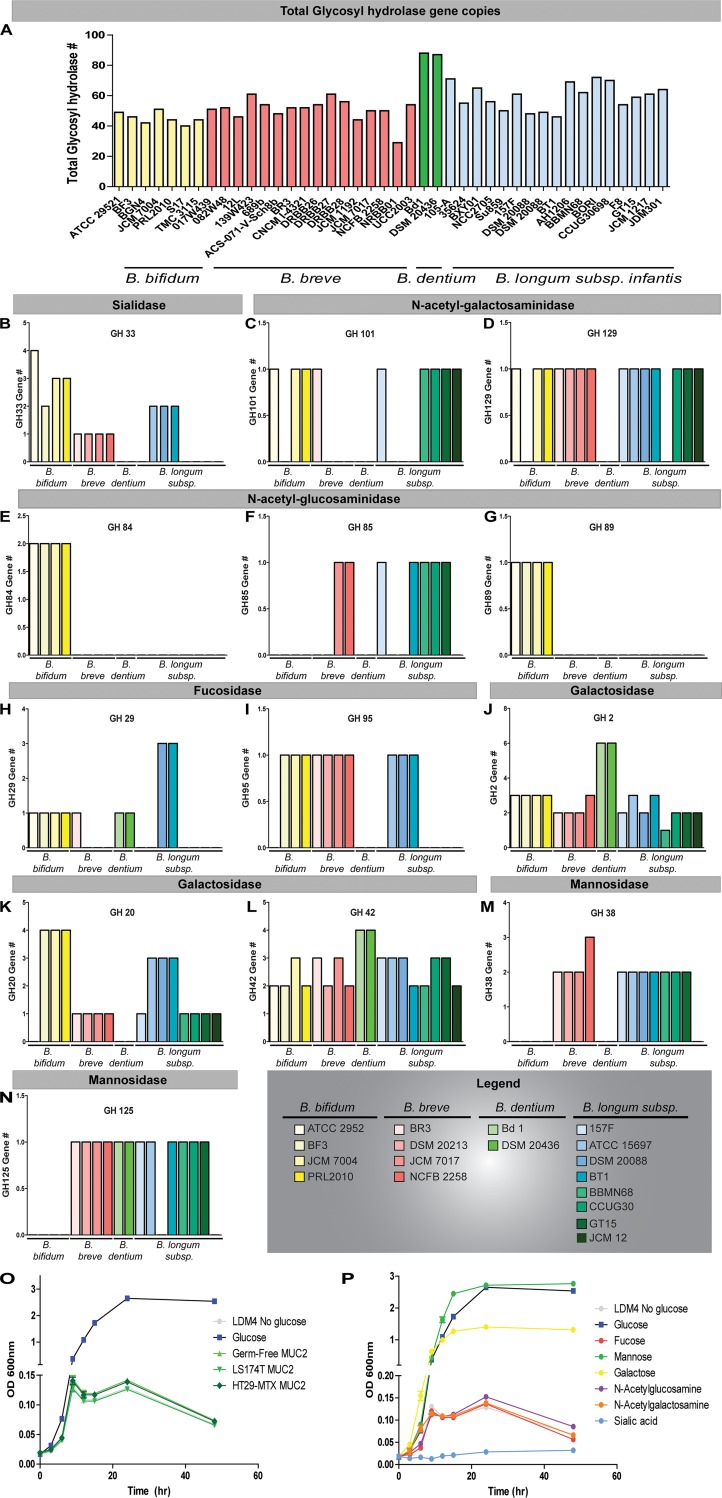
*B. dentium* harbors few glycosyl hydrolases and is unable to grow on Muc2 alone. Several bifidobacterial genomes were screened for glycosyl hydrolases using the CAZy database. (A to N) Data are presented in terms of copy number for glycosyl hydrolase (GH) families in the whole genome (A) and predicted to be involved in *O-*linked (B to L) and *N-*linked (M and N) mucin degradation. (O) *B. dentium* was grown in LDMIV with or without glucose or purified MUC2 from germfree cecal contents or human goblet cell line LS174T and HT29-MTX. (P) *B. dentium* was also grown without glucose in the presence of carbohydrates found in mucins. *n* = 4 groups/treatment, repeated 4 independent times. *, *P* < 0.05, one-way ANOVA.

A number of *Bifidobacterium* species harbor extensive glycosyl hydrolases (GHs) that are able to degrade mucin glycans (93). To define whether *B. dentium* was capable of degrading mucin glycans, we examined glycosyl hydrolases in *B. dentium* compared to glycosyl hydrolases across other *Bifidobacterium* species. *In silico* analysis of several *Bifidobacterium* genomes revealed that *B. dentium* genomes contain a greater number of glycosyl hydrolases (87.5 ± 0.7) than do strains of *B. bifidum* (45.1 ± 3.8), *B. breve* (50.9 ± 7.4), B. longum (59.4 ± 8.4), and B. longum subsp. *infantis* (59.6 ± 8.8) (Fig. 2A). Of these glycosyl hydrolases, only select families are involved in mucin degradation (Fig. 2B to N). The following glycosyl hydrolase families are involved in the degradation of mucin *O-*linked glycans: GH33 (sialidase), GH101 and GH129 (*N*-acetylgalactosaminidases), GH84, GH85, and GH89 (*N*-acetylglucosaminidases), GH20 (galactosidase), GH95 and GH29 (fucosidase), and GH2 and GH42 (galactosidase) (Fig. 2B to L). Additionally, the glycosyl hydrolase families GH38 and GH125 (mannosidases) are involved in *N-*linked glycan degradation (Fig. 2M and N). Our *in silico* analysis demonstrated the presence of a GH33 family member, which removes the terminal sialic acid residue, in several *Bifidobacterium* species (Fig. 2B). In contrast, the *B. dentium* Bd1 or DSM 20436 genomes do not encode any GH33 enzymes, indicating the inability to remove sialic acid. Some bifidobacteria also code for GH101 and GH129, which includes a cell wall-anchored endo-α-*N*-acetylgalactosaminidase that can remove entire glycan structures from the mucin protein (81, 94–98) (Fig. 2C and D). However, *B. dentium* did not harbor these glycosyl hydrolase families. We also detected *N-*acetylgalactosaminidases (GH84, GH85, and GH89) in several *Bifidobacterium* species, particularly the *B. bifidum* group (Fig. 2E to G). GH84, GH85, and GH89 were also absent in the *B. dentium* genome. Of note, *B. dentium* was predicted to remove α- and β-linked galactose (GH2 and GH42), α-fucose (GH29), and mannose (GH125) (Fig. 2G, I, K, and M). These findings indicate that *B. dentium* has a limited capacity to degrade mucin glycans.

Since *B. dentium* does not encode GH33, which removes terminal sialic acid groups and provides access to underlying carbohydrates in the glycan, we hypothesized that *B. dentium* would not be able to release carbohydrate residues from MUC2 and use them as a growth source. To address this question, *B. dentium* was grown in a fully defined medium (lactic acid bacteria defined medium IV [LDMIV]) with the carbon source (100 mM glucose) removed (Fig. 2O). Supplementation of purified germfree cecal MUC2 or MUC2 derived from the human mucin-producing cell lines LS174T or HT29-MTX at 1 mg/ml did not enhance *B. dentium* growth in LDMIV without glucose. Next, we examined whether *B. dentium* could grow in minimal medium in the presence of free oligosaccharides that are typically found in mucin (Fig. 2P). The addition of mannose and, to some degree, galactose was able to restore the growth of *B. dentium*. However, *B. dentium* was unable to grow on fucose, *N*-acetylglucosamine, *N-*acetylgalactosamine, or sialic acid as primary carbon sources. These data further support the idea that *B. dentium* does not extensively degrade mucin glycans and cannot use them as a fuel source.

### *B. dentium* colonizes the intestinal mucus layer *in vivo*.

**FIG 3:**
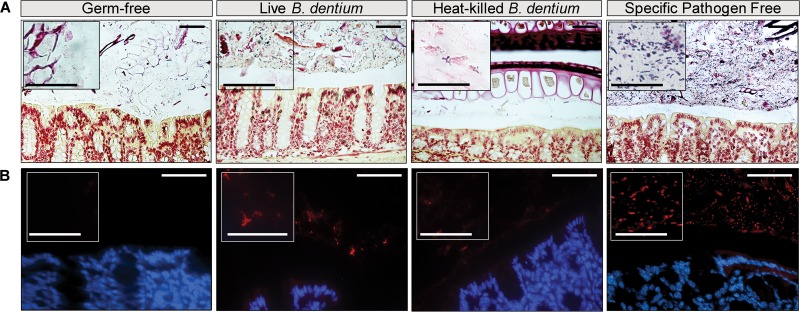
*B. dentium* colonizes gnotobiotic mouse colon. (A and B) Representative images of tissue Gram stains (×200 magnification, scale bar = 50 μm) (A) and fluorescence *in situ* hybridization (FISH) (×400 magnification, scale bar = 50 μm) (B) of the midcolon of germfree (*n* = 10), live *B. dentium*-monoassociated (*n* = 10), heat-killed *B. dentium*-treated (*n* = 10) and mice with a complete gut microbiota (specific pathogen free [SPF]) (*n* = 10). Insets reveal higher-magnification (×60) images of bacterial morphology.

Based on our *in vitro* data demonstrating that *B. dentium* adheres to purified MUC2 and existing literature demonstrating the mucus-modulatory effect of *Bifidobacterium* species, we next examined the effect of *B. dentium* on the intestinal mucus layer in a gnotobiotic model. Adult germfree Swiss Webster mice (male, *n* = 21; female, *n* = 23) were orally gavaged with either sterile de Man-Rogosa-Sharpe (MRS) medium, live *B. dentium* MRS cultures (2 × 10^8^ bacteria), or heat-killed *B. dentium* (2 × 10^8^ bacteria) every other day for 1 week. To prevent cross-contamination, mice were housed in separate isolators. For comparison, age-matched Swiss Webster specific-pathogen-free (SPF) mice that have a complex murine microbiota were included as controls. Microbe colonization was examined by tissue Gram staining, fluorescence *in situ* hybridization (FISH), quantitative real-time PCR (qPCR), and conventional plating on bacteriologic medium (Fig. 3). Gram stains of SPF mice indicate a robust microbial community residing in the mucus layer above the epithelium (Fig. 3A). Multiple bacterial morphologies were present in the SPF mice, including cocci and rods (Fig. 3A, inset). Microbial distribution was confirmed by FISH with a probe recognizing diverse bacteria (universal 16S rRNA gene-based probe) (Fig. 3B). In contrast to SPF mice, germfree mice exhibited no bacterial stains by Gram stain or FISH, while *B. dentium*-monoassociated mice exhibited classic bifid-shaped microbes in the mucus layer adjacent to the intestinal epithelium (Fig. 3A and B). Negligible bacterial staining was observed by Gram stain or FISH in mice treated with heat-killed *B. dentium* (Fig. 3A and B). Colonization of *B. dentium* was further confirmed using microbiologic plating of stool on MRS plates and qPCR of stool extract genomic DNA (gDNA) with *B. dentium*-specific primers. Data obtained by qPCR and microbial cultures with CFU resulted in calculations of approximately 7.2 × 10^7^ ± 1 × 10^7^ bacteria per gram in *B. dentium*-monoassociated mouse stool, with no observable CFU for germfree or heat-killed *B. dentium* groups. These findings indicate that live *B. dentium* can stably colonize the mucus layer of germfree mice.

**FIG 4:**
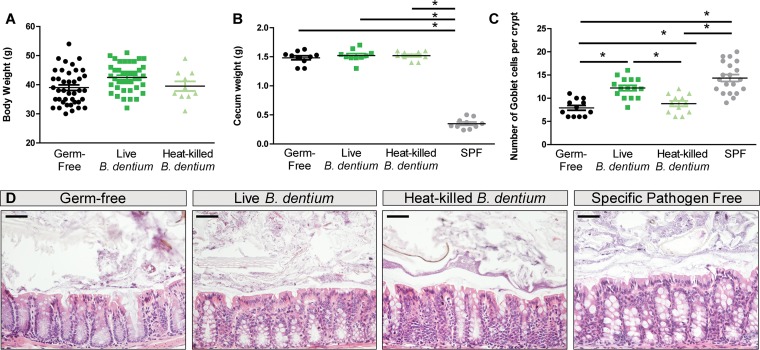
*B. dentium*-monoassociated mice yield increased numbers of intestinal goblet cells. (A) Weight measured at day 17 of germfree (black dots, *n* =40), live *B. dentium-*monoassociated (green dots, *n* =40), and heat-killed *B. dentium* treated (teal dots, *n* =10) mice. (B and C) Measurement of cecum weights (B) and quantification of goblet cells per colonic crypt by Fiji analysis (C) of germfree (*n* = 20 mice), live *B. dentium*-monoassociated (*n* = 20), heat-killed *B. dentium* (*n* = 10), and specific-pathogen-free (SPF) (*n* = 10) mice. All data are presented as averages of 3 images per section/mouse. (D) H&E stains (×200 magnification, scale bar = 50 μm) of midcolon from germfree, live *B. dentium*-monoassociated, heat-killed *B. dentium*-treated, and SPF mice. *n* = 10 mice/group. *, *P* < 0.05, one-way ANOVA.

Gut microbes are known to influence morphological parameters, including weight, cecum size, intestinal parameters, and development of the lamina propria (99–101). To determine if colonization by *B. dentium* influences overall mouse weight, mice were weighed on day 17 after monoassociation (Fig. 4A). No significant differences were observed in terms of mouse body mass between germfree, live, or heat-killed *B. dentium*-monoassociated groups (day 17 germfree, 40.5 ± 5.6 g; live *B. dentium*, 42.5 ± 4.8 g; heat-killed *B. dentium*, 39.5 ± 5.3 g). Germfree mice are characterized by an accumulation of mucins, proteins, and carbohydrates which results in abnormally large cecum size (102, 103). The morphologic difference between germfree and conventionally colonized mouse cecum is associated with the absence of mucus-degrading intestinal bacteria (104, 105). Cecal weights revealed that *B. dentium* monoassociation did not restore cecum size, as both germfree, live, and heat-killed *B. dentium*-colonized mice exhibited significantly longer ceca than their conventionally colonized counterparts (Fig. 4B). This finding is consistent with the prediction that *B. dentium* cannot extensively degrade mucins, and several species are likely to be necessary to modulate mucus sufficiently to affect cecum size.

To assess the effects of *B. dentium* colonization on intestinal morphology, we examined intestinal dimensions and architecture from hematoxylin and eosin (H&E) staining (Fig. 4C and D). No significant differences were observed for colon diameters or colon lengths between any of the groups (data not shown). H&E staining found no differences in crypt depths between germfree, live, or heat-killed *B. dentium*-colonized mice. However, H&E staining indicated that *B. dentium*-colonized mice exhibited an increase in filled goblet cell numbers per crypt relative to germfree mice and heat-killed *B. dentium*-treated mice (Fig. 4C and D).

### Goblet cells are modulated by *B. dentium* colonization.

**FIG 5:**
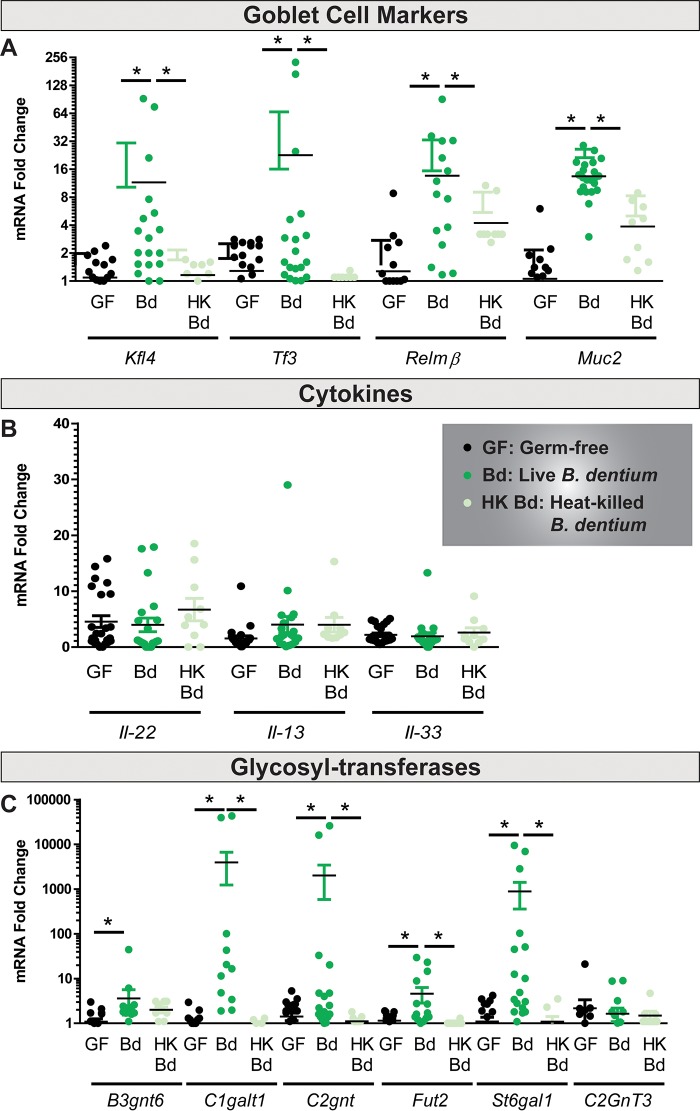
*B. dentium*-monoassociated mice have increased expression of goblet cell marker genes without corresponding changes in mucin-modulating cytokines. (A to C) qPCR analysis of germfree (black dots, *n* = 20), live *B. dentium*-monoassociated (green dots, *n* = 20), and heat-killed *B. dentium*-treated (teal dots, *n* = 10) colon for goblet cell markers (A), mucin-inducing cytokines (B), and goblet cell glycosyltransferase gene expression (C). All expression was normalized to GAPDH. *, *P* < 0.05, one-way ANOVA.

Next, we sought to define the effects of *B. dentium* monoassociation on goblet cell maturation and function. Compared to germfree mice and heat-killed *B. dentium* controls, *B. dentium*-colonized mice exhibited increased expression of Krüppel-like family of zinc-finger transcription factor 4 (*Klf4*), a goblet cell-specific differentiation factor (106) (Fig. 5A). Goblet cells are known to produce a number of important mucosal defense factors, such as resistin-like molecule-beta (*Relm*-β) and trefoil factor 3 (*Tff3*) (107). Both *Relm-B* and *Tff3* expression levels were increased in *B. dentium*-colonized colons compared with those of germfree or heat-killed *B. dentium* controls (Fig. 5A). MUC2 is the major gel-forming mucin protein secreted by intestinal goblet cells. Similar to other markers of goblet cells, *Muc2* mRNA was also increased in *B. dentium*-monoassociated mice compared with germfree and heat-killed *B. dentium* controls (Fig. 5A). Of note, a trend toward increased expression of *Relm*-β and *Muc2* was observed in heat-killed *B. dentium*-treated mice. Although not significant, this may point to a complementary role of *B. dentium* surface proteins in modulating goblet cells. Several cytokines are associated with increased mucus production, most notably interleukin-22 (IL-22), IL-33, and IL-13 (108–111). Interestingly, no changes were observed in any of these mucin-regulating cytokines (Fig. 5B), indicating that *B. dentium* may be modulating mucin production through secreted factors that affect the epithelium, rather than by stimulating the immune system.

### *B. dentium* modulates glycosylation of the intestinal mucus layer.

**FIG 6:**
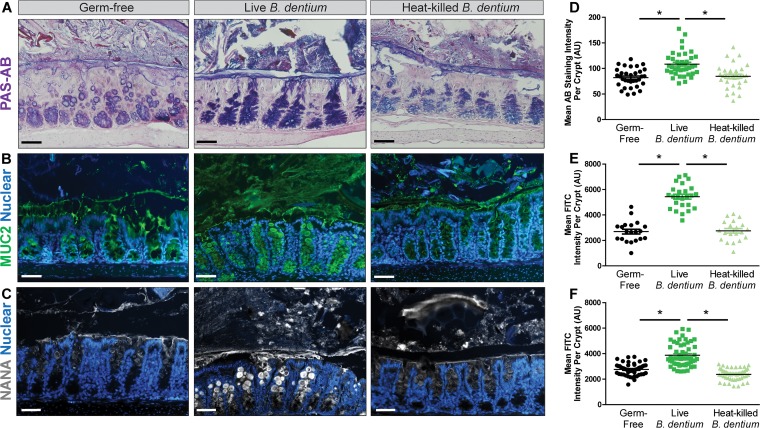
*B. dentium*-monoassociated mice have increased colonic Muc2 production. (A) Representative images (×200 magnification, scale bar = 50 μm) of PAS-AB stains of germfree, live *B. dentium*-monoassociated, and heat-killed *B. dentium* mouse midcolon. (B) Immunostaining of MUC2 (green) in mouse midcolon (×200 magnification, scale bar = 50 μm) counterstained with Hoechst. (C) FITC-labeled lectins (Vector Labs) were used to identify terminal carbohydrates, including sialic acid (also known as *N*-acetylneuraminic acid or NAN) (white) in mouse midcolon (×200 magnification, scale bar = 50 μm) counterstained with Hoechst. (D to F) Quantification of staining intensity per crypt or FITC fluorescence per crypt by Fiji. Fiji analysis was based on 5 representative images per mouse, *n* = 10 mice/group. *, *P* < 0.05, one-way ANOVA. AU, arbitrary units.

Mucin proteins are heavily *O-*glycosylated by glycosyltransferases within the goblet cell ER and Golgi. Glycosylation is influenced by colonization status, as intestinal bacteria are known drivers of mucin *O-*glycosylation (112–117). As a result, we examined changes in goblet cell glycosyltransferases in the presence of *B. dentium* (Fig. 5C). An examination of glycosyltransferases in *B. dentium*-monoassociated mice found significantly increased gene expression of several glycosyltransferases by qPCR compared with germfree controls. Increases in *B. dentium-*monoassociated colons compared with germfree controls were observed in the following glycosyltransferases: *B3gnt6* (β-1,3-*N*-acetylglucosaminyltransferase 6), *C1galt1* (β-1,3-galactosyltransferase 1), *C2gnt* [glucosaminyl (*N*-acetyl)transferase 1], *Fut2* (fucosyltransferase 2), *C2GnT1* [glucosaminyl (*N*-acetyl)transferase 1], and *St6gal1* (ST6 *N-*acetylgalactosaminide α-2,6-sialyltransferase). No change was observed in C2gnt3 [glucosaminyl (*N*-acetyl)transferase 3]. Increased St6gal1 was of interest, as it appends on terminal sialic acid residues to mucin glycans. The addition of sialic acid alters the charge of the mucus and enhances the potential for mucus to resist attack by bacterial enzymes (118, 119). Mirroring the increased expression of *Muc2*, *B. dentium-*treated mice also had increased periodic acid-Schiff–alcian blue (PAS-AB)-positive goblet cells, which identifies negatively charged mucins compared with germfree and heat-killed *B. dentium*-treated mice (Fig. 6A and D). Immunostaining corresponded with the PAS-AB results and demonstrated increased MUC2 protein in *B. dentium*-monoassociated mice compared with germfree and heat-killed *B. dentium* controls (Fig. 6B and E), indicating that live *B. dentium* can upregulate MUC2 *in vivo*. An examination of terminal mucin sugars using lectins revealed no differences between the terminal sugars fucose, mannose, galactose, *N-*acetyl*-* d-glucosamine (GlcNAc), *N*-acetyl-d-galactosamine (GalNAc) in *B. dentium*-colonized mice (live or dead), and germfree mice (data not shown). Consistent with increased St6gal1, which appends on sialic acid, *B. dentium*-colonized mice exhibited increased amounts of α-2,6-linked sialic acid in intestinal mucins compared with germfree and heat-killed *B. dentium* controls (Fig. 6C and F). These results indicate that *B. dentium* alone cannot degrade terminal sialic acid and thus cannot efficiently degrade the mucus layer.

### *B. dentium* stimulates autophagy-mediated Ca^2+^ signaling and mucus expulsion.

**FIG 7:**
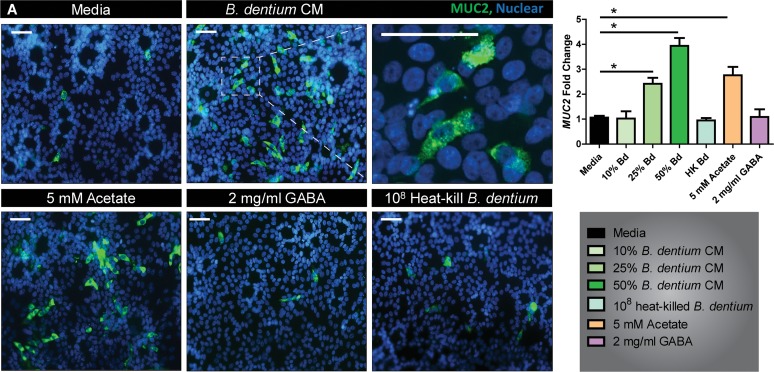
*B. dentium* secretes products, including acetate, that increase human Muc2 production. (A) Immunostaining of MUC2 (green) and nuclear dye Hoechst (blue) in T84 cells treated with *B. dentium*-conditioned medium, 5 mM acetate, 2 mg/ml GABA, or 10^8^ heat-killed *B. dentium* bacteria (×200 magnification, all scale bars [including magnified inset] = 50 μm). (B) qPCR analysis of *MUC2* gene expression in response to increased concentrations of *B. dentium*-conditioned medium and acetate. *n* = 6 to 8 wells of T84/treatment group, with 3 independent experiments. *, *P* < 0.05, one-way ANOVA.

Based on our *in vivo* data, which demonstrated significant changes in MUC2 production in response to live *B. dentium*, we speculated that secreted factors play a role in modulating goblet cell function. Similar to other bifidobacteria, the *B. dentium* genome harbors the machinery required to generate acetate (65, 120) (KEGG Pathway, map 01120), a short-chain fatty acid that has also been shown to stimulate *Muc2* expression (121, 122). We have also previously shown that *B. dentium* secretes 3.4 ± 0.4 mg/ml GABA (87), and other groups have found that activation of GABA type A (GABA_A_) receptors stimulates the release of stored mucin granules (123). To determine if *B. dentium* secretes factors that increase MUC2 synthesis, we utilized the T84 intestinal epithelial cell line, which can form goblet-like cells with MUC2-containing secretory granules (124). T84 cells harbor GABA receptors (125, 126) and an acetate receptor (GPR43) (127), allowing us to examine the effects of these compounds on MUC2 levels. The addition of *B. dentium*-conditioned medium resulted in increased MUC2 by immunostaining compared to medium controls (Fig. 7A). Moreover, the addition of acetate, but not GABA or heat-killed *B. dentium*, increased MUC2 staining. At the mRNA level, the addition of increasing concentrations of *B. dentium*-conditioned medium, as well as 5 mM acetate, resulted in increased levels of *MUC2* (Fig. 7B). This result indicates that *B. dentium* can secrete products, such as acetate, that are capable of increasing *MUC2* synthesis.

**FIG 8:**
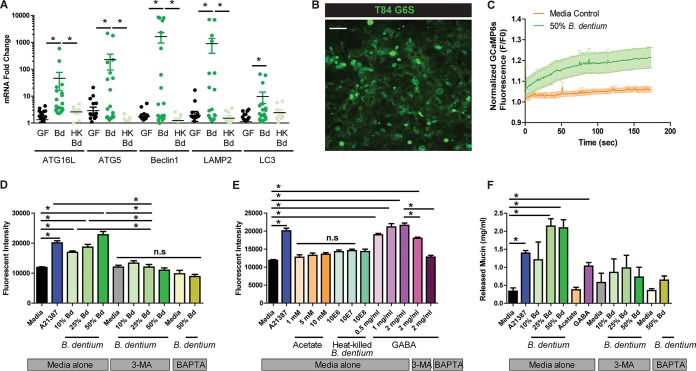
*B. dentium* activates autophagy and stimulates calcium signaling. (A) qPCR analysis of autophagy genes in germfree mice (black dots, *n* = 20), live *B. dentium*-monoassociated mice (green dots, *n* = 20), and heat-killed *B. dentium*-treated mice (*n* = 10 mice/group). (B) T84 cells transduced with the green fluorescent protein (GFP)-based calcium sensor GCaMP6s (×200 magnification). (C) Quantification of live imaging of T84 cells in response to medium (control) and *B. dentium* FluoroBrite conditioned DMEM over 3 h. *n* = 6 to 8 wells of T84/treatment group, with 3 independent experiments. (D and E) Release of CFDA-SE-tagged proteins measured at 1 h in response to *B. dentium* (Bd)-conditioned medium, heat-killed *B. dentium*, acetate, or GABA and inhibitors (calcium quencher BAPTA-AM and autophagy inhibitor 3-MA) or activators (calcium ionophore A21387). Fluorescent intensity determined by fluorescence absorbance at excitation 488 and emission 528. (F) Mucin content was measured in the supernatant by PAS-AB quantification using purified Muc2 as a standard. *n* = 8 wells of T84/treatment group, for 3 independent experiments. *, *P* < 0.05, one-way ANOVA; n.s., nonsignificant.

In addition to synthesizing MUC2, goblet cells release stored MUC2 granules in response to the proper stimuli. Work by Patel et al. has demonstrated that autophagy is required to elicit Ca^2+^ signaling within intestinal goblet cells (128), and that defects in autophagy (ATG5 deficiency) result in a loss of Ca^2+^ mobilization and accumulation of mucin granules within goblet cells. In parallel with these findings, multiple studies have found that Ca^2+^ signaling is required for the release of mucin-filled vacuoles (128–131). We examined a number of known autophagy markers by qPCR and found that *B. dentium* colonization was associated with increased expression of *ATG16L*, *ATG5*, *Beclin1*, *LAMP2*, and *LC3* (Fig. 8A). To determine if *B. dentium* was capable of eliciting Ca^2+^ signaling and MUC2 release, we first created mucin-producing T84 cells that stably expressed the cytoplasmic genetically encoded Ca^2+^ indicator GCaMP6s using lentivirus transduction (132). We then incubated the T84 GCaMP6s cells with *B. dentium* FluoroBrite conditioned Dulbecco's modified Eagle medium (DMEM) and measured the relative cytoplasmic Ca^2+^ concentration using epifluorescence microscopy (Fig. 8B and C). The addition of *B. dentium*-conditioned medium significantly increased Ca^2+^ levels (Fig. 8C). Mucin release was quantified by the release of CFDA-SE-tagged proteins at 1 h (Fig. 8D and E) or by alcian blue staining of T84 cell supernatants after 24 h (Fig. 8F). Corresponding to Ca^2+^ signaling data, *B. dentium*-conditioned medium elicited mucin release (Fig. 8D to F). The results obtained with *B. dentium* conditioned medium (CM) were comparable to results obtained with calcium ionophore A21387. Notably, the intracellular calcium chelator BAPTA-AM [1,2-bis(2-aminophenoxy)ethane-*N*,*N*,*N*′,*N*′-tetraacetic acid tetrakis(acetoxymethyl ester)] prevented the secretion of mucin. Moreover, pretreatment with the autophagy inhibitor 3-methyladenine (3-MA) inhibited *B. dentium* stimulated-mucus release, indicating that *B. dentium* requires both autophagy and Ca^2+^ mobilization to promote mucin secretion (Fig. 8D and F). We also demonstrated that GABA stimulates mucin expulsion, an effect which can be partially inhibited by 3-MA and completely inhibited by BAPTA-AM (Fig. 8E and F). Acetate and heat-killed *B. dentium* had no effect on mucin expulsion (Fig. 8E and F), indicating that *B. dentium*-secreted products, including GABA, are capable of stimulating the release of MUC2 from goblet cells. Together, this work provides evidence that *B. dentium* is unable to degrade mucin glycans or proteins to an appreciable extent to support growth but can stimulate host mucin production and secretion to bolster the mucus barrier. This work highlights the unique nature of *B. dentium* as a “mucin builder” and potential therapeutic for mucin-related disorders.

## DISCUSSION

Herein, we describe the first gnotobiotic mouse model with a mucin-building, human-derived *Bifidobacterium* species, *B. dentium*. By studying monoassociated mouse models with human gut microbes adept at modulating intestinal mucus, we have gained new insights into the microbe-mammal interface. We demonstrate that *B. dentium* adheres to intestinal mucus and colonizes the mucus layer *in vivo*, but it does not harbor the genetic machinery required to significantly degrade the mucin glycans. We demonstrate that the presence of *B. dentium* correlates with significant increases in mucin gene expression as well as increased quantities of MUC2 protein. *B. dentium*-secreted products, such as acetate, were able to increase MUC2 production *in vitro. B. dentium* also releases products which regulate intestinal goblet cells, including GABA, which stimulates autophagy-mediated Ca^2+^ signaling and MUC2 release. These findings are supported by our *in vivo* findings of increased autophagy gene expression in *B. dentium*-colonized mice. We postulate that these factors work together in concert to mediate the mucin-building effects of *B. dentium*. Together, these findings suggest that *B. dentium* may be a desirable mucin builder *Bifidobacterium* species for the treatment of mucin-related disorders.

Our work is the first to provide conclusive evidence of *Bifidobacterium* species directly modulating MUC2 and glycosylation *in vivo*. Bifidobacteria are known to colonize the intestinal mucus layer, and this location has been speculated to allow for the optimal delivery of microbial metabolites to promote mucin production (43, 44, 59–61, 63, 133–135). Several studies point to the role of bifidobacteria in mucin maintenance in rats and mice with a complex gut microbiota (24–27, 136). The contributions of specific gut microbes is difficult to decipher in SPF animals, making the development of gnotobiotic mouse models attractive for studies of microbiome-mucus layer biology. Our work demonstrates that *B. dentium* is able to adhere to intestinal MUC2 and expands prior studies by reporting that *B. dentium* produces compounds that stimulate MUC2 synthesis. Our *in vivo* gnotobiotic mouse model and our *in vitro* T84 human cell model support this conclusion. In support of a specific role of secreted metabolites in stimulating mucus production, we found no differences in the cytokines known to stimulate intestinal mucus production (108, 109, 137–140). Additionally, our data using heat-killed *B. dentium* indicate that *B. dentium* surface components do not play a large role in modulating MUC2 *in vivo* or *in vitro*. Although lipoteichoic acids (LTA) from Gram-positive bacteria and other Toll-like receptor (TLR) agonists have been reported to modulate mucin production (141–147), we did not observe significant changes in MUC2 levels in response to *B. dentium* surface proteins. Single-cell sequencing indicates that mouse goblet cells exhibit low TLR2 expression compared to other cell types (Single Cell Portal; Broad Institute). We speculate that this observation may account for our lack of significant goblet cell modulation by our heat-killed bacteria. Our *in vitro* work in human T84 cells also indicated that heat-killed *B. dentium* has no effect of MUC2 synthesis or expulsion. This finding may be due to our T84 cell line model, and further studies are needed to assess *B. dentium*-mediated TLR activation in human goblet cells.

Based on our data, we speculate that *B. dentium* enhances mucus production via the secretion of microbial metabolites such as acetate and GABA. *B. dentium* does not contain the genes responsible for generating butyrate or propionate, but it is capable of synthesizing acetate (KEGG). As such, we speculate that the MUC2-modulating effects of *B. dentium* may be due in part to acetate production. Acetate may work through multiple pathways. According to single-cell sequencing of mouse intestinal epithelium, the acetate receptor GPR43 (also known as FFAR2) is highly expressed in goblet cells (Single Cell Portal; Broad Institute), and acetate may directly modulate GPR43 to stimulate MUC2 production. Additionally, acetate is known to lower the intestinal pH (148, 149), and the organization of MUC2 in goblet cells is triggered by low pH (150). Moreover, enteroendocrine cells in the intestine can be stimulated by low pH or GPR43 activation to secrete serotonin, which is also known to influence goblet cells (151–154). In addition to acetate, it is possible that *B. dentium* secretes other products that regulate MUC2 production. Another lactic acid-producing bacterium, Lactobacillus rhamnosus GG, secretes a 40-kDa protein, termed p40, which has 27 to 78% homology with CHAP domain-containing proteins secreted by various *Bifidobacterium* strains, including *B. dentium* (155). These compounds may also be involved in *B. dentium*-mediated mucin modulation.

In addition to promoting MUC2 synthesis, we demonstrate that *B. dentium* metabolites stimulate autophagy-mediated Ca^+2^ signaling and mucin release. MUC2 secretion and the renewal of the colonic mucus layer are essential for the protective function of mucins. Goblet cells can release stored mucin granules in response to autophagy and Ca^2+^-mobilizing agents, such as acetylcholine or other cholinergic agonists (144, 156–161). Patel et al. found that autophagy was required for mucin release, and ATG5-deficient mice were unable to release MUC2 (128). Moreover, another autophagy-deficient mouse model, ATG7 knockout mice, exhibited a diminished mucus layer and was more susceptible to dextran sulfate sodium (DSS)-induced colitis (161). We are the first to link bacterium-promoted autophagy with goblet cell mucin expulsion. Our work shows that *B. dentium* can stimulate autophagy-driven Ca^2+^ signaling. We also demonstrate that GABA is capable of eliciting mucin secretion, an effect which can be minimized by blocking the autophagy pathway by 3-MA. Interestingly, RELM-β, a compound secreted by goblet cells, has also been shown to act as a mucin secretagogue in the mouse colon and HT29-Cl.16E human gut cell lines (162). RELM-β is upregulated in response to *B. dentium* colonization in our model and may serve to further promote MUC2 expulsion *in vivo*.

Finally, we demonstrate that *B. dentium* is unique among several well-characterized human *Bifidobacterium* strains, as it is unable to extensively degrade mucin glycans. Although the CAZy database does not have GHs annotated for our *B. dentium* ATCC 27678 strain, a BioCyc Database Basic Local Alignment Search Tool (BLAST) search shows 99 to 100% homology between mucin-degrading GHs in *B. dentium* ATCC 27678 and *B. dentium* Bd1 (UniProt D2Q7E7 [GH29], D2Q5B6 [GH125], D2Q767 [GH2], and D2Q935 [GH42]), indicating that the Bd1 genome can be used as a proxy for ATCC 27678 analysis. Based on *in silico* analysis and *in vitro* experiments, *Bifidobacterium* species do not produce mucinases that can cleave the MUC2 mucin protein core (163). However, many bifidobacteria do synthesize mucin carbohydrate-cleaving glycosyl hydrolases (65, 164). These human gut species include Bifidobacterium scardovii, B. longum subsp. longum, B. longum subsp. *infantis, B. breve*, and *B. bifidum* (165). *B. dentium* does not contain genes of the GH33 sialidase family or genes of the GH101 or GH129 families that enable cleavage of mucin glycans from the mucin protein. Despite the fact that the *B. dentium* genome contains more GHs than the average *Bifidobacterium* species (165), the *B. dentium* genome only contains a limited assortment of mucin-related GH genes (GH2, GH42, GH29, and GH125). The majority of the *B. dentium* GH enzymes are predicted to utilize and convert various intracellular or target dietary compounds, such as cellobiose, xylose, ribose, arabinose, mannitol, and sorbitol (136). Interestingly, the glycosyl hydrolases harbored by *B. dentium* may be advantageous in early life. *B. dentium* is frequently isolated from healthy breast-fed infants (39, 84, 85, 166). Breast milk glycans commonly contain galactose residues (167), and *B. dentium* harbors the galactosidases GH2 and GH42. As a result, *B. dentium* may use milk glycans during postnatal human development to establish a niche.

Consistent with the larger repertoire of mucin-degrading glycosyl hydrolases, previous studies have shown that mucin-maintaining or mucin-degrading species, such as *B. bifidum* (PRL2010, D119, and L22), *B. breve* NCIMB8807, and B. longum NCIMB8809, can grow in the presence of mucin alone (72, 75, 168–170). Our data indicate that *B. dentium* does not harbor the same glycosyl hydrolase genes and is unable to grow in mucin alone. Under pathological conditions with a diminished gastrointestinal mucus layer, a relative preponderance of mucin builders versus mucin degraders may be beneficial. Mucin-building bifidobacteria that enhance mucin production without mucin-degrading abilities would be preferred under specific biological conditions. *B. dentium* has the distinct ability to upregulate MUC2 synthesis, influence host glycosylation patterns, and promote MUC2 secretion, coupled with the inability to degrade mammalian host mucins. Thus, we speculate that *B. dentium* may be beneficial in intestinal environments lacking sufficient mucin.

One caveat to our work is that several bifidobacterial species have been identified in dental caries (120, 171–181). These bifidobacteria include gut-associated strains, such as those of *B. breve*, *B. adolescentis*, B. longum, and our microbe of interest, *B. dentium*. It has been postulated that bifidobacteria exist as stable species in dental caries due to their adhesive properties and their resistance to acidity (173, 182, 183). However, the results of clinical studies assessing the effects of bifidobacteria on the oral microbiota are controversial. Currently, no studies have dissected the role of bifidobacteria in the pathogenesis of caries. Of note, studies have also shown that bifidobacteria, including *B. dentium*, inhibit the growth of Porphyromonas gingivalis in *in vitro* biofilm models (184), which may have a positive effect on subgingival biofilm and thereby may enhance gingival health. One theory, the specific plaque hypothesis, has proposed that only a few specific species, such as Streptococcus mutans and Streptococcus sobrinus, are actively involved in the disease (185). While it is possible that *Bifidobacterium* species may be indirectly contributing to caries via acid production, the role of bifidobacteria within these carries remains unclear. More studies are needed before it is possible to draw conclusions on bifidobacteria in dental caries.

*B. dentium*, similar to other *Bifidobacterium* strains, is a recognized member of the healthy infant and adult human gut microbiota, according to the Human Microbiome Project (86) and sequencing studies (39, 84, 85, 166). Our data indicate that *B. dentium* is a commensal bacterium of the intestine and harbors beneficial mucin-modulating properties. Collectively, these data point to the potential role of next-generation mucin-building probiotics, such as *B. dentium*, in rescuing intestinal mucus layer function and treating intestinal disorders in the future.

## MATERIALS AND METHODS

### Materials.

The monosaccharides d-glucose and d-mannose were purchased from Sigma-Aldrich. l-Fucose, *N-*acetyl*-* d-glucosamine (GlcNAc), *N*-acetyl-d-galactosamine (GalNAc), and *N-*acetylneuraminic acid (Neu5Ac) were purchased from Carbosynth Limited. All other reagents were from Sigma-Aldrich, unless otherwise stated.

### Bacteriology.

Bifidobacterium dentium ATCC 27678 (adult human fecal isolate), Bifidobacterium longum subsp. *infantis* ATCC 15697 (infant intestinal isolate), Bifidobacterium longum subsp. *longum* ATCC 55813 (adult human fecal isolate), and Bifidobacterium breve ATCC 15698 (infant intestinal isolate) (ATCC, American Type Culture Collection) were cultured in an anaerobic workstation (Anaerobe Systems AS-580) in a mixture of 5% CO_2_, 5% H_2_, and 90% N_2._ Colonies were grown in de Man-Rogosa-Sharpe (MRS) medium (Difco) anaerobically at 37°C overnight. Single colonies were isolated from cultures plated on MRS agar plates. *B. dentium* was adjusted to optical density at 600 nm (OD_600_) of 0.1, subcultured into a fully defined medium termed lactic acid bacteria defined medium IV (LDMIV) (see Table S1 in the supplemental material), and incubated for up to 48 h anaerobically at 37°C. Bacterial growth was measured by the OD_600_ and CFU counts on MRS agar. For T84 cell treatment, *B. dentium* was grown overnight in MRS anaerobically at 37°C, and cells were pelleted by centrifugation at 5,000 × *g* for 5 min. Bacterial cells were washed three times with sterile anaerobic phosphate-buffered saline (PBS) to wash away residual MRS, and the bacterial pellet was resuspended in anaerobic optically clear FluoroBrite DMEM (catalog no. A1896701; Thermo Fisher) and incubated for 6 h. Following the incubation, cultures were centrifuged to remove bacteria, the pH was adjusted to 7, and the cultures were filtered through a 0.2-μm-pore-size polyvinylidene difluoride (PVDF) membrane (Millipore) to sterilize the supernatant, termed “conditioned medium.” For animal experiments, *B. dentium* was grown anaerobically in MRS to log-phase growth (1.6 × 10^9^ cells ml^−1^). Bacterial viability was confirmed for each gavage session by serially plating *B. dentium* and assessing CFU via conventional plating on MRS agar.

10.1128/mBio.01087-19.1 TABLE S1 Recipe for the fully defined lactic acid bacteria defined medium IV. Download Table S1, PDF file, 0.10 MB. Copyright © 2019 Engevik et al. 2019 Engevik et al. This content is distributed under the terms of the Creative Commons Attribution 4.0 International license.

### Gnotobiotic mouse model.

All experimental procedures and animal care were approved by the Institutional Animal Care and Use Committee (IACUC) at Baylor College of Medicine, Houston, TX. Swiss Webster germfree mice were purchased from Taconic and housed in the Baylor College of Medicine germfree facility. Mice were housed in filter-top cages in sterile isolators, and all food, water, and bedding were irradiated for sterility. Feces from experimental mice and isolator sentinels were collected at various time points and examined routinely for colonization by plating anaerobically and aerobically on blood agar. Male and female adult 7- to 9-month-old mice were housed under gnotobiotic (germfree) conditions. Germfree mice were monoassociated by oral gavage with 3.2 × 10^8^ CFU ml^−1^ *B. dentium* ATCC 27678 grown in MRS (total, *n* = 33; males, *n* =16; females, *n* =17). Control germfree mice received sterile MRS gavages (total, *n* = 31; males, *n* = 14; females, *n* =17) or 3.2 × 10^8^ CFU ml^−1^ heat-killed *B. dentium* (males, *n* = 5; females, *n* = 5). For heat-killed bacteria, *B. dentium* was heated at 60°C for 30 min. Culture viability was confirmed by plating on MRS agar.

Cultures were maintained in sterile Hungate tubes until administration to the mice to maintain anaerobic conditions. Mice received oral gavage doses once every other day for 1 week and one final gavage a week later. Fecal samples were plated on MRS and blood agar (Hardy Diagnostics) over time to monitor colonization. Plates were incubated anaerobically and aerobically at 37°C. To ensure that the germfree mice harbored no bacteria, gDNA was extracted from stool samples before the start of the experiment and at the time of euthanasia and examined by qPCR using a 16S rRNA gene-based universal bacterial probe for bacterial colonization. Weight was monitored over the course of treatment, and mice were euthanized 72 h after the last gavage.

### Intestinal tissue staining.

**(i) Immunofluorescence.** Mouse tissue segments were embedded in paraffin, and 7-μm sections were processed for staining. Following dehydration, slides were incubated for 20 min at 100°C in antigen unmasking solution citrate buffer pH 6 (Vector Labs) in a steamer for antigen retrieval. Sections were blocked for 1 h at room temperature in 10% donkey and/or goat serum. Staining was performed with an anti-MUC2 antibody (dilution 1:200, rabbit anti-MUC2, catalog no. sc-15334, Santa Cruz Biotechnology Inc.) and incubated overnight at 4°C. The primary antibody was recognized using donkey-anti-rabbit Alexa Fluor 488 antibody diluted at 1:300 (Life Technologies) and incubated for 1 h at room temperature. Hoechst 33342 (Invitrogen) was incubated at room temperature for 10 min to stain the nuclei. Coverslips were mounted with mounting medium (Life Technologies).

A panel of fluorescein isothiocyanate (FITC)-conjugated lectins were used to identify terminal mucin glycans. These lectins included Ulex europaeus agglutinin-1 (UEA-1; sugar recognized, fucose), concanavalin A agglutinin (CONA; mannose), Dolichos biflorus agglutinin (DBA; *N*-acetylgalactosamine), peanut agglutinin (PNA; galactose), wheat germ agglutinin (WGA; *N*-acetylglucosamine), and Sambucus nigra agglutinin (SNA; sialic acid) (catalog no. FLK-2100 [for UEA-1, CONA, DBA, PNA, WGA] and FL-1201 [for SNA]; Vector Laboratories), as previously described (5). Briefly, deparaffinized sections were blocked with PBS containing 10% bovine albumin (BSA) and stained with 10 μg/ml FITC-labeled lectin at room temperature for 1 h. Sections were washed three times in PBS and counterstained in Hoechst 33342. All slides were imaged on an upright wide-field epifluorescence Nikon Eclipse 90i microscope with the following objectives: 20× Plan Apo (numerical aperture [NA], 0.75) differential interference contrast (DIC) objective and a 40× Plan Apo (NA, 0.95) DIC objective. All fluorescent images were recorded using a Cool*SNAP* HQ2 camera (Photometrics) with a Spectra X light-emitting diode (LED) light source (Lumencor). Semiquantitative analysis of fluorescent stains was accomplished using Fiji (formerly known as ImageJ) software by tabulating the mean pixel intensity (National Institutes of Health) in five regions/per slide, at *n* = 11 to 12 mice/group, as previously described (4, 186, 187).

**(ii) H&E, PAS-AB, and tissue Gram stain.** Paraffin-embedded tissue sections were serially dehydrated in xylene and ethanol. Sections were stained with hematoxylin and eosin (H&E) for intestine architecture or periodic acid-Schiff–alcian blue (PAS-AB) to identify goblet cells and mucus. To identify bacterial colonization, sections were incubated with the following Gram staining reagents: crystal violet, Gram iodine, and safranin, with a series of washes with a decolorizer solvent, followed by picric acid-acetone. After alcohol and xylene dehydration steps, sections were coverslipped with Fluoromount aqueous mounting medium (Sigma-Aldrich catalog no. F4680) and imaged on an upright wide-field Nikon Eclipse 90i microscope. H&E and PAS-AB sections were recorded using a DS-Fi1-U2 camera (Nikon) with a 20× Plan Apo (NA, 0.75) DIC objective.

**(iii) Fluorescence *in situ* hybridization staining.** *B. dentium* localization was examined in mouse colon sections using the *Bifidobacterium*-specific FISH probe Bif164 (5′-CATCCGGCATTACCACCC-3′), and total bacteria were examined using the universal bacterial FISH probe EUB338 (5′-GCTGCCTCCCGTAGGAGT-3′) (Integrated DNA Technologies [IDT]). Brieﬂy, 7-μm sections were dehydrated in ethanol and incubated with the *Bifidobacterium* probe at 45°C in a dark humidifying chamber. Slides were hybridized for 45 min and counterstained with Hoechst 33342 for 10 min at room temperature (Invitrogen). All slides were imaged on an upright wide-field epifluorescence Nikon Eclipse 90i microscope (Tokyo, Japan) with a 40× Plan Apo (NA, 0.95) DIC objective. FISH images were recorded using a Cool*SNAP* HQ2 camera (Photometrics) using a Nikon Intensilight C-HGFI mercury lamp.

### Germfree mouse mucus isolation.

Germfree mouse cecum contents were collected (*n* = 7) and pooled for mucus extraction. Mucus was prepared similarly to previously described methods (70, 91). Briefly, pooled cecum samples were diluted in four volumes of ice-cold phosphate-buffered saline containing EDTA, phenylmethylsulfonyl fluoride, and iodoacetamide. Particulate matter was removed by centrifugation, and mucin protein was isolated by precipitation with ice-cold ethanol. Precipitated mucin was lyophilized and purified by CsCl isopycnic density gradient centrifugation at a starting density of 1.39 g ml^−1^ using a 70 Ti rotor (Beckman Coulter, Inc.) (188, 189). Dialyzed mucins were lyophilized and resuspended in HEPES-buffered Hanks’ salt solution (HH) at a concentration of 1 mg/ml. Mucin concentration was ascertained by a bicinchoninic acid (BCA) assay with purified pig stomach mucin (Sigma-Aldrich) as a standard. For adhesion assays, 100 μl of mucus solution (1 mg/ml) was immobilized in 96-well polystyrene microtiter plates (Maxisorp, Nunc; VWR) by overnight incubation at 4°C, as previously described (70). Wells were washed 3× with 200 μl of HH prior to the addition of bacteria to remove unbound mucus.

### Mucin adhesion assay.

Bifidobacterium dentium ATCC 27678, Bifidobacterium infantis ATCC 15697, Bifidobacterium breve ATCC 15698, and Bifidobacterium longum ATCC 55813 were grown anaerobically in MRS. Bacterial suspensions were adjusted to an OD_600_ of 2 in PBS and fluorescently tagged by incubating with 10 μM carboxyfluorescein diacetate-succinimidyl ester (CFDA-SE; catalog no. C1157; Thermo Fisher) for 30 min at 37°C anaerobically, similar to previous bacterial staining reports (190, 191). Fluorescent bacteria were washed 3 times with anaerobic PBS and resuspended in PBS at an OD_600_ of 2. To determine bacterial adhesion, 100 μl of labeled bacteria was added to the microtiter plates coated with germfree mouse mucus and 2-fold serially diluted in PBS. Following a 1-h incubation at 37°C, the wells were washed 3 times with 200 μl of HH to remove unattached bacteria. Bacterial adherence was examined by reading fluorescence (excitation/emission, 492/517 nm) in a microtiter plate reader (Synergy H1; Biotek), and the presence of bacteria was confirmed by microscopy. The results from the adhesion assay are presented as means from four replicates in three independent experiments.

### T84 culturing and treatment.

**(i) T84 cell culturing.** Human colon-derived T84 (ATCC CCL-248) cells were obtained from the ATCC. Cells were regularly maintained in a complete growth medium (CGM) of Gibco Dulbecco's modified Eagle medium F-12 (Thermo Fisher) supplemented with 2 mM GlutaMAX (Thermo Fisher) and 10% fetal bovine serum (FBS). Cultures were maintained in a humidified atmosphere at 37°C and 5% CO_2_. Cells were routinely tested for Mycoplasma contamination using the Mycoplasma detection kit (catalog no. LT07-518; Lonza). For an examination of *Muc2* mRNA levels, cells were seeded at 5 × 10^4^ cells/cm^2^ in 12-well tissue culture-treated plates (Corning) until the cells reached confluence. Cells were then treated with various concentrations of *B. dentium* FluoroBrite conditioned DMEM, heat-killed *B. dentium* bacteria, γ-aminobutyric acid (GABA), or 5 mM sodium acetate overnight in DMEM without glucose and without FBS. Following incubation (16 to 18 h), cells were treated with TRIzol reagent for RNA extraction.

**(ii) T84 mucin expulsion assays.** T84 cells were seeded at 5 × 10^4^ cells/cm^2^ into a 24-well plate and incubated at 37°C and 5% CO_2_ until the cells reached confluence. Cells were then serum starved overnight and treated with various amounts of *B. dentium* FluoroBrite conditioned DMEM, GABA, or acetate diluted in FluoroBrite DMEM with 1× GlutaMAX (no FBS). To inhibit autophagy, cells were pretreated for 1 h with the PI3K inhibitor 3-methyladenine (3-MA; Sigma-Aldrich catalog no. M9281). As positive and negative controls, cells were treated with 10 μM the calcium ionophore A21387, a known stimulator of mucus (124), or 25 μM BAPTA, a Ca^2+^ chelator which is known to inhibit mucus production (192, 193). Supernatant (100 μl) was collected at 1, 3, and 24 h and incubated with alcian blue, as previously described (194). Briefly, the supernatant was incubated with an equal volume of 1% alcian blue and 3% glacial acetic acid for 2 h at room temperature. Mucins were pelleted by centrifugation at 12,000 × *g* for 10 min, and pellets were washed three times with PBS. Following washes, mucin pellets were resuspended in PBS containing 10% SDS and sonicated to release the alcian blue color. Absorbance was measured at 620 nm on a spectrophotometer. As an alternative method for analyzing mucus release, confluent T84 monolayers in 96-well plates were thoroughly washed with PBS and incubated with 10 μM CFDA-SE in PBS for 30 min at 37°C and 5% CO_2_ to fluorescently tag all proteins. Following the incubation, cells were washed three times in PBS and incubated in FluoroBrite DMEM for 3 h at 37°C and 5% CO_2_ to allow the CFDA-SE to incorporate into all proteins. Cells were then treated with *B. dentium* FluoroBrite conditioned DMEM, GABA, acetate, 3-MA, A21387, or BAPTA, as described above. Supernatants (100 μl) were collected at 1 h and examined by fluorescent plate reader at an excitation/emission of 492/517 nm.

**(iii) Generation of T84 GCaMP6S cells for live-cell calcium imaging.** For calcium (Ca^2+^) imaging studies, T84 cells were cultured in 12-well plates to 50 to 60% confluence and transduced using lentivirus pLVX-GCaMP6s with 10 μg/ml Polybrene (catalog no. TR-1003-G; Millipore) in CGM and incubated for 48 h. The lentivirus construct and packaging were previously described (195). Two days after transduction, cells were cultured in the presence of puromycin (10 μg/ml) to select for stably expressing cells. For Ca^2+^ measurements, T84 GCaMP6s were grown to confluence on 8-well chamber slides (Matek) and then changed to an optically clear FluoroBrite DMEM (Invitrogen) supplemented with 15 mM HEPES (Invitrogen), 1× sodium pyruvate (Invitrogen), 1× GlutaMAX (Invitrogen), and 1× nonessential amino acids (Invitrogen). *B. dentium* FluoroBrite conditioned DMEM was added in uninoculated FluoroBrite DMEM (50% final concentration). Cells were placed in an Okolabs stage-top incubation chamber with CO_2_ mixing and humidity control and placed on an Nikon TiE inverted wide-field epifluorescence microscope with a motorized X, Y, and Z stage for software-controlled multiposition imaging. Cells were imaged with wide-field epifluorescence using a 20× PlanFluor (NA, 0.45) phase-contrast objective or a 20× Plan Apo (NA, 0.75) differential interference contrast objective, using a Spectra X LED light source (Lumencor). Images were recorded using an ORCA-Flash 4.0 scientific complementary metal-oxide semiconductor (sCMOS) camera (Hamamatsu), and the Nikon Elements v4.5 software was used for data acquisition and image analysis. Following the experiment, cell viability was examined by staining cells with 3 μM calcein AM (live-cell dye, catalog no. C3099; Invitrogen) and 2.5 μM propidium iodide (dead-cell dye, catalog no. P4170; Sigma-Aldrich).

### Scanning electron microscopy.

The MUC2-producing human colonic carcinoma cell line LS174T ATCC CL-188 (ATCC) was grown in DMEM supplemented with 10% fetal bovine serum at 37°C and 5% CO_2_. Approximately 1 × 10^5^ cells were seeded onto Corning Costar 24-well culture plates containing poly-l-lysine-coated coverslips and grown to confluence. *B. dentium* was incubated with confluent coverslips at 2 × 10^5^ bacteria and incubated for 1 h anaerobically at 37°C. Coverslips were then washed thoroughly with PBS (3 times) and fixed in 2.5% glutaraldehyde in PBS for 1 h at room temperature. Coverslips were dehydrated with ethanol and coated in 20 nm of gold using a desktop sputtering system (Denton Desk II). Slides were viewed in a scanning electron microscope (FEI XL-30FEG) at 12 kV.

### Quantitative real-time PCR.

A stool sample was collected to examine bacterial composition, and genomic DNA was isolated using the Zymo gDNA MiniPrep kit (catalog no. 11-317C; Zymo Research) with bead beating. Mouse colon cells were stored in TRIzol reagent, and RNA was extracted according to the manufacturer’s details. The SensiFAST cDNA synthesis kit (Bioline USA, Inc.) was used to synthesize cDNA from 1 μg RNA. cDNA was examined by quantitative real-time PCR (qPCR) via Fast SYBR green (Thermo Fisher) and primers (Table 1) using a QuantStudio 3 qPCR machine (Applied Biosystems). All reactions were carried out in 96-well plates with melting curves to ensure primer specificity. Relative mRNA levels were examined using the ΔΔ*CT* method with the glyceraldehyde-3-phosphate dehydrogenase (GAPDH) housekeeping gene, generating fold changes for each gene.

**TABLE 1:** Primer sequences for real-time PCR using SYBR green chemistry

Gene target	Species	Primer sequence (5′–3′)		Reference or source
		Forward	Reverse	
*Muc2*	Mouse	TGCCCACCTCCTCAAAGAC	TAGTTTCCGTTGGAACAGTGAA	12
*Klf4*	Mouse	AGGAACTCTCTCACATGAAGCG	GGTCGTTGAACTCCTCGGTC	NCBI
*Relm-*β	Mouse	CCATTTCCTGAGCTTTCTGG	AGCACATCCAGTGACAACCA	107
*Tff3*	Mouse	CAGATTACGTTGGCCTGTCTCC	ATGCTTGCTACCCTTGGACCAC	107
*IL-22*	Mouse	TTGAGGTGTCCAACTTCCAGCA	AGCCGGACGTCTGTGTTGTTA	196
*IL-13*	Mouse	AGACCAGACTCCCCTGTGCA	TGGGTCCTGTAGATGGCATTG	197
*IL-33*	Mouse	TCC TTG CTT GGC AGT ATC CA	TGC TCA ATG TGT CAA CAG ACG	198
*C2GnT3*	Mouse	AGGCTCCTCTTCCCTCAAAG	ACATCACCGTCCTCCAAGTC	199
*B3gnt6*	Mouse	CTTCGCGCCTTATGAGATG	CCTGTTTGTGGCTACAGTGC	116
*St6gal1*	Mouse	TGAGCCTTCCCCAAATACCT	TTCACAGGATGATCAAAAACCA	116
*Fut3*	Mouse	CCTGTCCCACGCAGTCTC	GAAACCAACAAAGCCTTGGA	116
*C1galt1*	Mouse	TGGAATTACAACTATTATCCTCCCATA	CAACATAGTGAAAAGAAACTGCGATA	116
*C2gnt*	Mouse	GCAGCCAAGAAGGTACCAAA	ACAGGCGAGGACCATCAA	116
*Fut-1*	Mouse	CAGCTCTGCCTGACATTTCTG	AGCAGGTGATAGTCTGAACACA	200
*Fut-2*	Mouse	AGTCTTCGTGGTTACAAGCAAC	TGGCTGGTGAGCCCTCAATA	200
*MUC2*	Human	CTGCACCAAGACCGTCCTCATG	GCAAGGACTGAACAAAGACTCAGA	201

### Statistical analysis.

Graphs were generated using the GraphPad Prism software (version 6; GraphPad, Inc.). For statistical analysis, comparisons were made with either one-way analysis of variance (ANOVA) with the Holm-Sidak *post hoc* test or with a *t* test using SigmaPlot. The data are presented as mean ± standard deviation, with differences between the groups considered significant at a *P* value of <0.05.
