# Supplementary material for: Bifidobacterium dentium Fortifies the Intestinal Mucus Layer via Autophagy and Calcium Signaling Pathways
Source: mBio. 2019 Jun 18;10(3):e01087-19. doi: 10.1128/mBio.01087-19 (PMC6581858; doi:10.1128/mBio.01087-19)
Supplement: TABLE S1 [file mBio.01087-19-st001.pdf]

Supplemental Table 1: Lactic Acid Bacteria Defined Medium (LDM) IV

Medium Base:

Ingredient	Amount	Catalog #
**Potassium phosphate dibasic trihydrate**	1.3 g	Sigma P5504
**Potassium phosphate monobasic**	5.0 g	Sigma P5655
**Sodium acetate**	1.0 g	Sigma S5636
**Ammonium citrate tribasic**	0.8 g	Sigma A1332
**L-Ascorbic acid**	0.5 g	Sigma 255564
**Sodium chloride**	1.2 g	Sigma S5886
**L- Alanine**	0.24 g	Sigma A7469
**L-Arginine**	0.125 g	Sigma A8094
**Asparagine**	0.2 g	Sigma A4284
**Aspartic Acid**	0.42 g	Sigma A8949
**L-Cysteine HCl monohydrate**	0.2 g	VWR A1038922
**L-Glutamic Acid**	0.5 g	Fluka 49450
**Glycine**	0.175 g	Sigma G8790
**L-Histidine**	0.15 g	Sigma H8000
**L-Isoleucine**	0.21 g	Sigma I7403
**L-Leucine**	0.475 g	Sigma L8912
**L-Lysine monohydrochloride**	0.55 g	Sigma L8662
**L-Methionine**	0.125 g	Sigma M5308
**Phenylalanine**	0.275 g	Sigma P5482
**Proline**	0.675 g	Sigma P5607
**Serine**	0.34 g	Sigma S4311
**Threonine**	0.225 g	Sigma T8441
**Tryptophan**	0.05 g	Sigma T8941
**Tyrosine**	0.25 g	Sigma T8566
**Valine**	0.325 g	Sigma V0513

Dissolve components in 937.5 ml of water. Autoclave at 121°C for 22 minutes. Add other solutions to this base.

Vitamin Solution (2000x in water)

Ingredient	Amount	Catalog #
**Thiamin hydrochloride**	0.01 g	Sigma T3902
**4-aminobenzoic acid**	0.002 g	Sigma A9878
**D-Panthothenic acid hemicalcium salt**	0.02 g	Sigma P2250
**Niacin (Nicotinic acid)**	0.05 g	Sigma N0761
**Pyridoxine hydrochloride**	0.025 g	Sigma P6280

Dissolve in 25 ml water. Filter sterilize. Store at 4°C for 6-8 weeks.

Biotin Solution (2000x in 0.01M HCl)

Ingredient	Amount	Catalog #
**Biotin**	0.005 g	Sigma B4501
**1 M Hydrochloric acid**	0.5 mL	Sigma H9892

Add HCl to 49.5 mL water, then add biotin. Add a few drops of 95% ethanol to dissolve, if necessary. Filter sterilize. Store at 4°C.

Riboflavin Solution (200x in 0.02M Acetic Acid)

Ingredient	Amount	Catalog #
**Riboflavin**	0.004 g	Sigma R9504
**Acetic acid (99-100%)**	0.057 mL	Sigma 27221

Add acetic acid to 49.943 mL water, then add riboflavin. Heat to 40°C to dissolve, if necessary. Filter sterilize. Store at 4°C.
Protect from light.

Folic Acid Solution (2000x in 0.01 M NaOH)

Ingredient	Amount	Catalog #
**Folic Acid**	0.01 g	Sigma F8758
**Sodium Hydroxide**	0.02 g	Sigma 8045

Dissolve in 50 ml water. Filter sterilize. Store at 4°C.

Nucleic Acid Solution I (333x in 1M HCl)

Ingredient	Amount	Catalog #
**Adenine sulfate**	0.05 g	Sigma A3159
**Guanine hydrochloride**	0.05 g	USB 16740
**Cytidine 5'-monohosphate**	0.1 g	Sigma C1131
**1 M Hydrochloric acid**	15 ml	Sigma H9892

Dissolve in 15 ml 1M HCl. Filter sterilize. Store at 4°C. Note: If visible crystals are seen in this solution after storage, prepare a fresh stock.

Nucleic Acid Solution II (1000x in 1M NaOH)

Ingredient	Amount	Catalog #
**Uracil**	0.2 g	Sigma U1128
**NaOH**	0.4 g	Sigma S8045

Dissolve in 10 ml water. Filter sterilize. Store at 4°C.

Nucleic Acid Solution III (1000x in water)

Ingredient	Amount	Catalog #
**Thymidine**	0.02 g	Sigma T1895

Dissolve in 12.5 ml water. Filter sterilize. Store at 4°C.

Salt Solution (1000x in water)

Ingredient	Amount	Catalog #
**Magnesium sulfate heptahydrate**	1.625 g	Sigma M1880
**Manganese sulfate monohydrate**	0.143 g	Sigma M7899
**Ferrous sulfate heptahydrate**	0.130 g	Sigma F8048

Dissolve in 10 ml water. Filter sterilize. Store at 4°C for 1-3 days. Note: If the solution turns yellow, discard and prepare a new one.

Glucose Solution (1000x in water)

Ingredient	Amount	Catalog #
**D-Glucose**	200 g	Sigma G8270

Dissolve in 400 mL water, bring final volume to 500 mL. Filter sterilize. Store at 4°C.

Final medium:

Ingredient	Amount
**Glucose solution**	50 mL
**Vitamin solution**	0.5 mL
**Biotin solution**	0.5 mL
**Riboflavin solution**	5 mL
**Folic acid solution**	0.5 mL
**Nucleic acid solution I**	3 mL
**Nucleic acid solution II**	1 mL
**Nucleic acid solution III**	1 mL
**Salt solution**	1 mL

Add solutions above to base solution in 1 liter bottle. Measure pH and adjust to 6.5 if necessary. Re-filter sterilize if necessary. Store final solution at 4°C, protected from light for 6-8 weeks
